# A tale of two systems: poisoning management in Iran and the United States

**DOI:** 10.1186/2008-2231-21-42

**Published:** 2013-05-29

**Authors:** Omid Mehrpour, Nasim Zamani, Jeffrey Brent, Mohammad Abdollahi

**Affiliations:** 1 Birjand Atherosclerosis and Coronary Artery Research Center, Birjand University of Medical Science, Birjand, Iran; 2 Medical Toxicology and Drug Abuse Research Center (MTDRC), Birjand University of Medical Sciences (BUMS), Pasdaran Avenue, Birjand 9713643138, Iran; 3 Department of Clinical Toxicology, Loghman Hakim Hospital, Shahid Beheshti University of Medical Sciences, Tehran, Iran; 4 University of Colorado, School of Medicine, Denver, CO, USA; 5 Department of Toxicology and Pharmacology, Faculty of Pharmacy, and Pharmaceutical Sciences Research Center, Tehran University of Medical Sciences (TUMS), Tehran 1417614411, Iran

**Keywords:** Iran, Poisoning Management, Review, USA

## Abstract

Poisoning morbidity and mortality is high in the developing world. Systems for care of poisoned patients differ markedly between countries. In this paper a comparison of two very different systems for the care of poisoned patients, is presented. Specifically, the role of poison centers and poison treatment centers in the US and Iran are contrasted. A systematic literature search was undertaken utilizing the PubMed, Scopus, and Google Scholar and the keywords “poison centers”, “treatment” “Iran” “United States of America” and 100 publications were identified. From these, relevant data were found in 23 publications. The information was double-checked and data were summarized herein.

We find that the system of the care of poisoned patients relies heavily on certified poison centers in the US and that only a few hospitals have well developed medical toxicology services. In contrast, in Iran, the poison center system is somehow less developed and the care of poisoned patients is provided in centralized high volume hospital poison units.

Although both the US and Iran have highly developed systems for the care of poisoned patients they are distinctly different. Comparative studies based on these systems could provide important data for developing countries with more rudimentary poison control and treatment facilities.

## Introduction

Poisoning is a very common cause of hospital admissions and even death worldwide. It has been reported to be the cause of death in as many as 593000 people in the developing world annually (intentional and unintentional) [1]. It is one of the most common causes of mortality and morbidity in countries such as Bangladesh and India. Every day in the United States (US), an average of 87 people are reported to die as a result of unintentional poisoning, and another 2277 are treated in emergency departments (EDs) [2]. In 2005, intentional and unintentional poisonings led to $33.4 billion in medical and productivity costs in the US [3].

In Iran no such statistics exist. However, clinical toxicologists confront many intoxicated patients in the few specific poison treatment centers in Iran every day and it is expected that the mortality rate due to poisoning may be as many as 8 and 209 deaths for each 1,000 ward-admitted and 1,000 ICU-admitted patients, respectively. In fact, in Iran, the most common reason for hospitalization, and the second cause of mortality in hospitalized patients, is poisoning [4].

Although poisoning is a problem for all countries, its profile of age, intention, and clinical presentation can differ substantially. Similarly, there are profound differences in the systems for the management of poisoned patients in various countries. In developing countries, all poisoned patients tend to be evaluated primarily in EDs, with hospital admission if necessary. In contrast, in European countries and the US, there has been an active system of poison centers for at least 5 decades [5]. These centers allow active initial contact by telephone and provide specialized advice by physicians, pharmacists and nurses with specific training in clinical toxicology. However, US poison centers do not provide bedside patient care. Rather, if hospitalization or ED evaluation is required, they triage patients to local hospitals. In contrast, only a small number of mostly tertiary care centers in the US have medical toxicology services. Given this difference in the ways in which poisoned patients are managed, we set out to evaluate the methods of triaging and treating poisoned patients in Iran and compare it with the way these patients are handled in the US. It is hoped that a comparison of these two very different systems can lead to subsequent evaluations of the strengths and weaknesses of each and hence produce hypotheses on the optimal method of triaging and poisoned patients.

## Methods

PubMed central, Scopus, Google, and Google Scholar were searched for the articles about poison treatment centers in USA and Iran. Our keywords were “poison centers”, “treatment”, “Iran”, and “United States of America”. A total of 100 articles were retrieved by the searches. Those from prior to 1995 were excluded. Of the remainder, 23 were judged to be compatible with our study aims. These studies were evaluated and relevant data was abstracted and double checked by one of authors and verified by two others.

### Structure of poisoning treatment centers

Almost 40,000 physicians work in Iran; of them 30–50 are Medical Toxicologists (MTs). The MTs in Iran are physicians who have mostly studied Toxicology, Anesthesiology, Pediatrics, Internal Medicine, Forensic Medicine, and Emergency Medicine in their residency period and/or after the residency, they have passed a clinical toxicology fellowship program. They are mainly allocated to one of ten hospitals in Iran specialized in the management of these poisonings [4]. The largest poisoning treatment centers of Iran are located at Loghman Hakim Hospital of Tehran and Imam Reza Hospital of Mashhad. Loghman Hakim Hospital’s poison center as the largest one provides services to as many as 25,000 poisoned patients (in- or out-patient) a year [6]. Besides to Loghman Hakim Hospital of Tehran and Imam Reza Hospital of Mashhad, other hospitals skilled in the management of the poisoned patients are Baharloo Hospital in Tehran, Noor Hospital in Isfahan, Razi Hospital in Ahwaz, Farshchian Hospital in Hamadan, and Ali-Asghar Hospital in Shiraz, and some private clinics, etc. [6]. In addition, poisoning management centers in Birjand, Arak, Ghom, Ardabil, Tabriz, Sari and Rasht are developing [6].

In the US, there is only one center with a specialized unit and allocated beds for the treatment of poisoned patients. That center is located in Harrisburg, Pennsylvania, and is known as the Pinnacle Health Toxicology Center. The bulk of the initial contact of poisoned patients with specialized toxicology care in the US comes from telephone contact with poison control centers. These centers then triage patients who require hospital evaluation to EDs of local Hospitals, most of which do not have specialized medical services. The current system of poison centers in the US relies on 5 components: 1. Regionalized poison information centers; 2. Training of poison center personnel and related health caregivers; 3. Utilization of poison information databases; 4. A national data collection system; and 5. A “systems approach” to care at a professional, state, and federal level.^7^ US Poison centers generally have three major goals: A. Primary prevention by activities such as identification of high frequency and low morbidity toxicities using their surveillance system, identification of the products that need protective packaging, and creation of necessary lay education programs; B. Secondary prevention by promoting public knowledge of the availability of the high quality poison centers, utilization of a uniform telephone number to call in the US, professional awareness of accurate data sources, well-trained professional staffing poison centers on a 24 hour per day basis, effective coordination with emergency medical services, and knowledge of the local availability for antidotes and specialized services such as hemodialysis; and C. Tertiary prevention by advising callers of interventions that minimize the severity of exposures, alerting emergency services, and triaging patients to hospitals if necessary [7].

In US, when a call is initiated to a poison center, the caller is quickly connected to a certified Specialist in Poison Information (CSPI) [8]. Professionals in US poison centers have access to poisoning databases such as Micromedex/Poisondex and consensus guidelines containing triage recommendations produced by the American Association of poison Control Centers (AAPC) published in Clinical Toxicology. This referral to hospitals is based on the substances to which the caller was exposed, the dose, and the caller’s symptoms [9]. One important goal of US poison centers is to determine when triage to a hospital is not indicated. Because inpatient or ED care in the US is very expensive, by keeping patients out of hospitals poison centers can potentially save significant amounts of money.

### Structure of poison control centers

Although there were some plans to develop a national toxicology information system as far back as 1985, the first official drug and poison information center (DPIC) was successfully established in Iran in 1995 [6] under the auspices of Ministry of Health and Medical Education and cooperation of nationwide Medical Universities [10]. Currently, there are about 30 DPICs in Iran located in various cities such as Tehran, Ahwaz, Ardabil, Isfahan, Karaj, Kerman, Khorramabad, Mashhad, Sanandaj, Shiraz, Tabriz, Yazd, etc., each of them covering a mean population of almost 2.5 million people [11]. Most of these centers are operative from 8 AM to 8 PM, however the one in Tehran stays open 24 hour a day, including all holidays. Most of DPICs are staffed with MD residents in toxicology and pharmacology or PharmD residents in toxicology, pharmacology, clinical pharmacy, or related sciences. The staff is available to offer advice to all callers with relating to drug information and poisoning cases. This information is available to both public and health professionals. Iranian centers also use caller information for toxico-surveillance. DPICs both provide answers to enquiries, and in cases of needing referral to EDs, these are made to hospitals with medical toxicology services. This kind of triaging is done to assure that poisoned patients receive the optimal possible care. DPICs also function to increase the knowledge of the health professionals and general public in various ways. To reach this objective, they use media and hold specific meetings in conjunction with major societies such as Iranian Society of Toxicology (IranTox).

In the USA, the story is far different. The first US poison center was established in Chicago in 1952. That event ushered in an area of proliferation of many other such centers. Their initial goal was to provide telephone services to the public. The orientation of these early centers was distinctly pediatric because most of their calls tended to involve potential exposures to children. Eventually a national network of such centers was developed under the auspices of the American Association of Poison Control Centers (AAPCC). That led to the establishment of a national data collection system. Originally known as the Toxic Exposure Surveillance System (TESS) [12], its most recent rendition is the National Poison Data System (“NPDS”). All US centers use electronic medical records while simultaneously uploading the data collection fields to the NPDS [8]. Over time, as poison centers have grown, the sophistication of their role has been expanded. This has been accomplished by the development of strict accreditation standards for poison centers and a certification examination for CSPIs. Although the latter are commonly registered nurses or pharmacists, some centers use individuals with other qualifications. US CSPIs must be recertified every 7 years [8]. Currently the primary goal of US poison centers is to give telephone advice to both the public and to health professionals. Other goals of US centers is to educate the public regarding poison prevention, detect and assist in the management of mass exposures, and to serve as a sentinel system to detect trends [13]. It is hoped that poison centers would reduce healthcare costs. To maintain their accreditation US poison centers must maintain strict standards and are reviewed every 5 years [8].

Today, 57 AAPCC certified poison centers exist in US. These centers were mostly located in the children’s Hospitals in the past and their directors were generally pediatricians. This was because of the concern for poisoning morbidity and mortality in the children.

In 1972, there were 216 reported fatal childhood poisoning cases in US [8]. This number has already declined to 39 children in 2007 [8]. Today about half of the calls to US poison centers involve pediatric cases [14]. In 2010, there were 3,952,722 encounters. Of these, 2,384,825 were human exposures, 94,823 calls were for animal exposures and 1,466,253 were classified as information calls [14]. US poison centers deal routinely with a great diversity of poisonings including those involving medications, illicit drug abuse, occupational exposures, and attempted self-harm by various means [8]. Virtually the entire US population has access to poison centers. In contrast, in an Iranian study, it was shown that between 2006 and 2008, DIPC of Loghman Hakim Hospital received a total of 9,694 calls [15].

### Identity and characteristics of the callers

According to the Iranian study, most of the callers to Iranian DIPCs are the patients’ relatives (49%) and are female (61%). Most patients are in the age range of 18 to 40 years. In 45.2% of the cases, the patients themselves called the DIPC. Most of the enquiries to Iranian poison centers involve questions about drug indications (24%) and adverse reactions (21.1%). The drugs most commonly involved are antidepressants (12.4%), antimicrobials (12%), and analgesics (11.2%). By providing answers to these enquiries the DIPCs provide an important public health service. However, medical caregivers’ calls are much less common [15]. Although the exact reason for the low rate of utilization of DPICs by medical professionals’ interest is not clear, Iranian physicians in general desire not to be involved in the treatment of poisoned patients. Rather they prefer to refer them to MTs. In contrast to the systems in Iran and the US, poison centers in many countries limit the calls they receive to healthcare providers and do not take calls from the public [8]. Unlike Iran, the US public and health professionals are both well acquainted with the availability of the poison center system.

### Information resources

In Iranian DIPCs, queries questions are answered by the use of several databases and books depending on their availability [15]. Almost similar resources are relied on by US poison centers. In addition, CSPIs always have access to physicians sub-specialty trained in MT for assistance with sick or complicated cases.

### National poison data system

The NPDS described above has no analogy in Iran. Setting up a system would be costly and labor intensive. Presently there is no available funding for doing this.

The US NPDS and its predecessor the TESS, function under the auspices of the AAPCC. Contained within this collective dataset is the experience of the US poison centers from 1985 to date. It has been reported that almost 42%, 83%, and 100% of the US population have been served by the poison centers in 1984, 1994, and 2003, respectively [12]. Over the years, the system has become more electronically-based and progressively more sophisticated. When someone calls a poison center in US, while giving needed information to the caller and management of the case, data are entered into the system by the CSPI using standardized AAPCC-mandated procedures. The data collected from the caller include case information, patient information (age, gender, and weight), exposure information, clinical symptoms, therapy strategies, and the outcome. The location of the caller is also determined by postal codes or telephone area code [12].

In the NPDS, the reason for exposure is classified as intentional, unintentional, adverse reaction to a drug or food, malicious exposure, suspected product contamination, or unknown. Clinical symptoms are classified using a 131-item list and include cardiovascular (signs and symptoms such as bradycardia, hematemesis, pneumonitis, hypoglycemia, etc.), dermal, gastrointestinal, hematologic/hepatic, neurologic, respiratory, ocular, renal, and miscellaneous. Therapies (n = 58) and decontamination methods (n = 10) are also noted and recorded. Medical outcome is ascertained and classified as no effect, minor effect, moderate effect, major effect, and death. Substances involved are categorized using a 7-digit coding system provided by the Poisindex database [12].

This AAPCC database has been shown to be effective in supporting regulatory actions such as child resistant closure on ethanol containing mouthwashes and re-classification of prescription medications to over-the-counter status. Hazards can be quantified in this database to calculate hazard factors (by determining the serious outcome per 1,000 exposures). However, the interpretation of these factors is limited by the bias implicit in the overall low morbidity of the caller population to US poison centers.

Every day, US poison centers collectively receive almost 11,000 calls. Therefore, the volume of cases reflected in the NPDS is huge. It is important to note that reports taken by US poison centers are not mandatory, come from many sources, are limited to the information provided by callers or obtained at callbacks, and are not verified. These factors limit the utility of the NPDS for scientific analyses and studies.

### Sources of support

In Iran, the expenses of DIPCs are generally provided by the Ministry of Health and Medical Education or the Universities in whose area the center is located. Because these centers use part-time personnel and do not support a national data collection system, costs are low. In contrast, maintaining the US system is costs approximately $136 million/year [16]. In the US, the costs of poison center operations is generally provided by state and local governments, Universities, Hospitals, charitable foundations, and the federal government. Currently, 12.6% of US poison center expenses are provided by US government. The US federal budget has a large deficit and in a cost cutting effort, there has been a 25% reduction in the federal funding for poison centers [8]. While the US is currently undergoing a transformation of its healthcare system, it is hoped that poison centers will continue to be part of federal efforts to reduce the costs and burden of poisonings to society [17].

As demonstrated over 15 years ago, the US public is willing to support poison centers [18]. Investigators from the California poison control system showed that by blocking calls to poison centers, the general population endorsed additional spending to continue to be served by these centers [18]. While the US population supports its poison centers, this is not the case in Iran, where patients simply prefer to go to hospitals without concern for the additional costs imposed on the health care system.

Restricting public access to poison control centers would result in additional costs to society, for several reasons, including inappropriate use of hospital facilities [19]. These data suggest that although the cost of poison centers is high, their cost-effectiveness justifies their existence. It is important to realize that the cost of healthcare in the US is very high, so even small savings can have a large impact. For example, the cost of an ED visit for a relatively minor poisoning with no required inpatient hospitalization could easily be in the range of $2,000 to 5,000. If a poison center can prevent that one visit, the cost savings to the individual and to society is large.

### Effectiveness of the services

The network of US poison centers has been successful in detecting the new poisoning outbreaks such as detection of the outbreak of fentanyl contaminated heroin and cyanide-laced acetaminophen, as well as episodes of food poisonings [8].

The network of 24 hours a day professionally staffed centers could be called into action for other types of public health activities and emergencies, for example when they assumed the role of information centers in diseases unrelated to poisoning such as during the recent outbreak of influenza H1N1, thus reducing ED costs of this disease dramatically [8]. The volume of calls to the US system renders it a unique sentinel detection system for natural or man-made outbreaks. When the calls from a unique area exceeds which would be normally expected in general, or related to a specific issue, an alert goes to MTs and to epidemiologists of the US CDC and AAPCC for monitoring. Since such an efficient cooperative system still does not exist in Iran, it is not expected that Iranian poison centers could fill this role.

### Antidotal therapy

The above data on poison center use in the US and in Iran may naturally raise the question of why these systems work so differently. The answer to this question is clearly multi-factorial. In the US, there is a widely promoted single telephone number that can be called from any place in country. Numerous public education campaigns, carried out both by local poison centers and the AAPCC encourage the use of these centers. US poison centers keep track of the availability of antidotes and capabilities in Hospitals in their region. In general, however, they tend to triage patients to the nearest hospital and most centers do not make an effort to refer specifically to facilities where there are MT services. If needed antidotes are not available at a particular hospital poison centers may advise that hospital regarding the nearest facilities where particular needed antidotes are available.

In contrast to the situation in the US, in Iran the system for the management of poisoned patients is highly centralized into specialized centers. Antidotes and trained MTs tend to be clustered at these centers. In 2009, antidotes were reported to be used 83,000 times in US [20]. According to the opinion of an expert consensus panel (established in 2009), 24 antidotes are recommended to be available in all Hospitals and another nine have been recommended to be available within a maximum of one hour [20]. Not all Hospitals in the US comply with these unofficial recommendations and it is here that the knowledge of antidote availability by poison centers becomes important. The same goes for Iran where maintaining a plentiful supply of antidotes at all hospitals is impossible at present. For instance, even some antidotes specified in the Iranian Drug List (IDL) such as calcium disodium edetate, ethanol, fomepizole, digoxin antigen binding fragment (Fab), mesoxalic acid, Prussian blue, and physostigmine sulfate have not been always available in most hospitals [4]. Approximately one quarter of the IDL registered antidotes are too expensive to be afforded by patients or their relatives and thus are rarely used in Iran. On the other hand, expensive antidotes such as fomepizole and Fabs are extensively used in US, even in suspected cases of methanol and digoxin toxicity, respectively. In Iran, the IDL-registered antidotes are generally found only in major referral hospitals serving as poison treatment centers in each province [20]. Lack of antidotes in some hospitals is one reason why some Iranian physicians are reluctant to manage poisoned patients and why they prefer to refer these patients to poison treatment centers. This also is one explanation for the few calls from health care professionals to the DIPCs. If antidote availability was increased, and professional education in MT was broadened, a system for the care of poisoned patients similar to that used in the US might be feasible. Doing so would decrease over-crowding of Iranian toxicology EDs. These changes, however, would require a major investment in the Iranian healthcare system.

### Toxicology training in Iran and USA

In both Iran and the US, there is an active system of continuing medical education programs in clinical toxicology [21]. The need for integration of MT education within undergraduate and postgraduate clinical training programs to maximize the care provided to poisoned patients has been recognized in Iran in the last decade. Therefore, training in MT is now being integrated into internships and in medical schools during the teaching of pathophysiology. In addition, fellowship training of MT has recently been established in some major poison treatment centers of the country. These training programs have demonstrated the popularity of MT with physicians, particularly specialists in Emergency Medicine, General Toxicology, Forensic Medicine, Pediatrics, and Internal Medicine, etc. [22]. Pharmacists or specialists of Toxicology are also actively participated in the CME courses.

In the US, MT fellowship training impacts the career of physicians by enhancing their academic opportunities and allowing them to develop MT services. Certification for physicians in MT in the US is technically under the purview of the specialty of Emergency Medicine, although physicians trained in other fields who complete an accredited MT fellowship may become sub-specialty certified. Currently, 63% of US Emergency Medicine training programs have a MT on the faculty in the US [22]. MT rotation is a requirement for just 76%, an elective at 19%, and not available at 5% of Emergency medicine programs. Specialties other than Emergency Medicine tend to have little formal training in MT as part of their curricula [22].

In general, although MT is highly developed in Iran compared to most other Middle Eastern countries [23], comparative analyses such as that presented herein and by others can be used as a springboard for ideas to promote academic and clinical development of MT [10,23]. Table 1 shows differences between the management options of the poisoned patients in Iran and USA.

**Table 1:** Differences between the management options of the poisoned patients in Iran and US

**Management options**		**Iran**	**US**
**Management centers**		**Mainly in toxicology hospitals**	**Mainly by poison info centers**
Poison control/information centers	Callers	Female/18- to 40-year-old patients/mostly self-harm cases poisoned by antidepressants	Female/children patients (52% of the calls)/mostly poisoned by illicit drugs and occupational exposures followed by self-harm exposures
	Responders	Mostly Residents of Toxicology or Clinical Pharmacy or Pharmacology and GP	Nurses or pharmacists specialist in poison information
	Information Resources	Mostly provided by books and Internet resources	Provided from the national poison database
	TESS	Absent	Present
	Costs	Not been evaluated yet	81 million dollars a year but proved to be cost-effective
Antidote availability		Poor	Although not quite compatible with standards, far better than Iran

Toxic Exposure Surveillance System (TESS); General physicians (GP).

## Competing interest

Both corresponding authors (JB and MA) are members of the AACT and cooperate in the international committee. The study was done in the line of scientific collaboration between IranTox and AACT.

## Authors’ contributions

OM and NZ drafted the manuscript. MA gave the idea. JB and MA provided advises and edited whole article. All authors read and approved the final manuscript.

## References

[B1] Akhlaghi M Arbabi A Khadivi R Pattern of acute poisoning in shahrekord (Western Iran) Asian J Epidemiol 2009 2 9 12 10.3923/aje.2009.9.12

[B2] Paulozzi LJ Weisler RH Patkar AA A national epidemic of unintentional prescription opioid overdose deaths: How physicians can help control it J Clin Psychiatry 2011 72 589 592 10.4088/JCP.10com06560 21536000

[B3] Poisoning in the United States: fact sheet Available at: [http://www.cdc.gov/homeandrecreationalsafety/Poisoning/poisoning-factsheet.htm] Accessed on 26 November 2012

[B4] Nikfar S Khatibi M Abdollahi-asl A Abdollahi M Cost and utilization study of antidotes Int J Pharmacol 2011 7 46 49

[B5] Roche L Vincent V [European organization of poison centers] Rev Lyon Med 1966 15 427 436 French 5943847

[B6] Mehrpour O Abdollahi M Poison treatment centers in Iran Hum Exp Toxicol 2012 31 303 304 10.1177/0960327110392086 21138986

[B7] Lovejoy FH Jr Robertson WO Woolf AD Poison centers, poison prevention, and the pediatrician Pediatrics 1994 94 220 224 8036077

[B8] Dart RC The secret life of America's poison centers Ann Emerg Med 2012 59 62 66 10.1016/j.annemergmed.2011.11.017 22177679

[B9] Benson BE Cheshire S McKinney PE Litovitz TL Tandberg D Foster H Do poison center guidelines adversely affect patient outcomes as triage referral values increase? J Toxicol Clin Toxicol 2003 41 585 590 10.1081/CLT-120023759 14514002

[B10] Pourmand A Wang J Mazer M A survey of poison control centers worldwide Daru 2012 20 13 10.1186/2008-2231-20-13 23351559 PMC3555748

[B11] Addresses poison centers 6 February 2012. Available at: [http://apps.who.int/poisoncentres/PC_addresses_20120206.pdf] Accessed on 26 November 2012

[B12] Watson WA Litovitz TL Belson MG Wolkin AB Patel M Schier JG Reid NE Kilbourne E Rubin C The Toxic Exposure Surveillance System (TESS): risk assessment and real-time toxicovigilance across United States poison centers Toxicol Appl Pharmacol 2005 207 604 610 10.1016/j.taap.2005.02.036 16023159

[B13] American College of Emergency Physicians (ACEP) Role of poison centers in emergency health care, preparedness, and response. Policy statement Ann Emerg Med 2011 57 314 21353920 10.1016/j.annemergmed.2010.11.019

[B14] Bronstein AC Spyker DA Cantilena LR Jr Green JL Rumack BH Dart RC 2010 annual report of the American Association of Poison Control Centers' National Poison Data System (NPDS): 28th annual report Clin Toxicol (Phila) 2011 49 910 941 10.3109/15563650.2011.635149 22165864

[B15] Shadnia S Soltaninejad K Sohrabi F Rezvani M Barari B Abdollahi M The performance of loghman-hakim drug and poison information center from 2006 to 2008 IJPR 2011 10 647 652 24250399 PMC3813026

[B16] Poison Centers Save More Than $1.8 Billion Every Year; New Report Quantifies the Value of the American Poison Center System Available at: [https://aapcc.s3.amazonaws.com/pdfs/releases/Lewin_Report_News_Release.pdf] Accessed on 26 November 2012

[B17] Giffin S Heard SE Budget cuts and U.S. Poison Centers - regional challenges create a nationwide problem Clin Toxicol (Phila) 2009 47 790 791 10.1080/15563650903250354 19778189

[B18] Phillips KA Homan RK Luft HS Hiatt PH Olson KR Kearney TE Heard SE Willingness to pay for poison control centers J Health Econ 1997 16 343 357 10.1016/S0167-6296(96)00521-8 10169305

[B19] Phillips KA Homan RK Hiatt PH Luft HS Kearney TE Heard SE Olson KR The costs and outcomes of restricting public access to poison control centers. Results from a natural experiment Med Care 1998 36 271 280 10.1097/00005650-199803000-00005 9520953

[B20] Shadnia S Garlich FM A comparison between the use and availability of antidotes in Iran and United States of America Int J Pharmacol 2011 7 799 800

[B21] Iranian society of toxicology Available at: [http://www.irantox.net/] Accessed on 10 January 2013

[B22] Medical toxicology training in Asia Pacific region Available at: [http://www.asiatox.org/8th%20apamt%20OP/17MEDICAL%20TOXICOLOGY%20TRAINING%20IN%20ASIA%20PACIFIC%20REGION.pdf]. Accessed on 26 November 2012

[B23] Brent J Abdollahi M The major role of toxicology societies in global collaborations-a call to action Daru 2012 20 11 10.1186/2008-2231-20-11 23351437 PMC3555728

